# Transverse effects on the nasomaxillary complex one year after rapid maxillary expansion as the only intervention: A controlled study

**DOI:** 10.1590/2176-9451.19.5.079-087.oar

**Published:** 2014

**Authors:** Carolina da Luz Baratieri, Maheus Alves, Claudia Trindade Mattos, Geórgia Wain Thi Lau, Lincoln Issamu Nojima, Margareth Maria Gomes de Souza

**Affiliations:** 1 Professor, Department of Orthodontics, Federal University of Santa Catarina (UFSC); 2 PhD resident, Federal University of Rio de Janeiro (UFRJ); 3 Adjunct professor, Fluminense Federal University (UFF)

**Keywords:** Palatal expansion technique, Longitudinal studies, Orthodontics, Cone-beam Computed Tomography

## Abstract

The aim of this study was to assess by means of cone-beam computed tomography (CBCT) scans the transverse effects on the nasomaxillary complex in patients submitted to rapid maxillary expansion (RME) using Haas expander in comparison to untreated individuals. This prospective controlled clinical study assessed 30 subjects (18 boys and 12 girls) with mixed dentition and during pubertal growth. The treated group was submitted to RME with Haas expander, retention for six months and a six-month follow-up after removal. The control group matched the treated group in terms of age and sex distribution. CBCT scans were taken at treatment onset and one year after the expander was activated. Maxillary first molars (U6) width, right and left U6 angulation, maxillary alveolar width, maxillary basal width, palatal alveolar width, palatal base width, right and left alveolar angulation, palatal area, nasal base width, nasal cavity width and inferior nasal cavity area on the posterior, middle and anterior coronal slices were measured with Dolphin Imaging Software^(r)^ 11.5, except for the first two variables which were performed only on the posterior slice. All transverse dimensions increased significantly (P < 0.05) in the treated group in comparison to the control, except for alveolar angulation and inferior nasal cavity area (P > 0.05). Results suggest that increase of molar, maxillary, palatal and nasal transverse dimensions was stable in comparison to the control group one year after treatment with RME.

## Abstract (pt)

**OBJETIVO:**

avaliar, por meio de tomografias computadorizada de feixe cônico (TCFC), as dimensões transversais do complexo nasomaxilar de pacientes submetidos à expansão rápida da maxila (ERM) e sem intervenção.

**MÉTODOS:**

este estudo clínico prospectivo e controlado avaliou 30 indivíduos (18 meninos e 12 meninas) durante o período de crescimento puberal e dentição mista. O grupo tratado foi submetido à ERM com expansor de Haas, permanecendo 6 meses em contenção e sendo acompanhados por mais 6 meses após a remoção. O grupo controle foi acompanhado durante o mesmo tempo. Foram realizadas TCFC ao início e um ano após a fase ativa. Com o programa Dolphin Imaging 11.5, a largura entre os primeiros molares superiores (U6), angulação dos U6 direito e esquerdo, largura maxilar alveolar, largura maxilar basal, largura alveolar do palato, largura basal do palato, angulação alveolar direita e esquerda, área palatina, largura da base nasal, largura da cavidade nasal e área da cavidade nasal inferior foram obtidas em cortes coronais posterior, médio e anterior, com exceção das duas primeiras medidas, que foram obtidas apenas no corte coronal posterior.

**RESULTADOS:**

todas as medidas foram significativamente (p < 0,05) maiores no grupo da ERM, com exceção da angulação alveolar e da área da cavidade nasal, as quais não foram significativas (p > 0,05) entre os grupos. Os resultados sugerem que, um ano após a ERM, a dimensão transversal na cavidade nasal, maxila, palato e região dos molares são maiores do que em indivíduos não tratados. O protocolo de ERM utilizado foi eficaz na manutenção da dimensão transversal durante período de acompanhamento.

## INTRODUCTION

Rapid maxillary expansion (RME) is the gold standard treatment for correction of maxillary transverse deficiency in primary, mixed and early permanent dentition.^1^ No other nonsurgical orthodontic intervention has greater impact on nasomaxillary development than this therapy when it is performed during the growth period. RME applies force on teeth and alveolar processes by activating the expansion screw and, as a result, promoting the opening of the midpalatal suture and widening the maxilla and its associated structures.^2^ Stability of the new transverse dimension is a fundamental part of this treatment, as it renders the retention phase as important as the active phase.

In general, clinical studies have assessed dental and skeletal effects immediately after RME or immediately after the retention period.^3^^,^ 4 However, the amount of relapse following a period without any retainer is still unclear. Cameron et al^5^ reported stability of increased maxillary and nasal widths 5 years after RME, comparing an experimental to a control group. Nevertheless, patients were also submitted to nonextraction edgewise treatment after the retention period of RME. It is not possible to assess the effects of RME alone if fixed orthodontic treatment is implemented, as the latter might cause transversal changes. Cone-beam computed tomography (CBCT) scans not only provide three-dimensional (3D) visualization of the craniofacial complex, but also allow accurate and reliable measurements of the changes promoted by RME, without image superimposition or distortion.^6^^,^ 7

The aim of this study was to assess by means of CBCT scans the transverse changes on the nasomaxillary complex of patients in mixed dentition submitted to RME with Haas expander. Their stability at one year after treatment was also assessed in comparison to a matched untreated control group.

## MATERIAL AND METHODS

This prospective controlled clinical study was approved by the Federal University of Rio de Janeiro Institutional Review Board (0044.0.239.000-11). It assessed 30 subjects (18 males and 12 females with mean age of 9 years and 4 months for men and 9 years and 7 months for women), admitted in the Department of Orthodontics of the Federal University of Rio de Janeiro. An informed consent form was signed by all parents and/or guardians. In selecting the sample, the following inclusion criteria were applied: Early mixed dentition; Class I or II malocclusions; patients at a stage prior to the pubertal growth peak (the stages of skeletal maturity were CS1, CS2 or CS3, as evaluated by the Cervical Vertebral Maturation method);^8^ no systemic diseases; and healthy dentition. Subjects who needed RME therapy were included in the treated group, as they presented posterior transverse interarch discrepancy measured as the difference between maxillary and mandibular intermolar widths.^9^ Maxillary skeletal transverse deficiency (distance from the J point to the facial frontal line < 12 mm)^10^ was confirmed for all subjects comprising the treated group.

**Figure 1:**
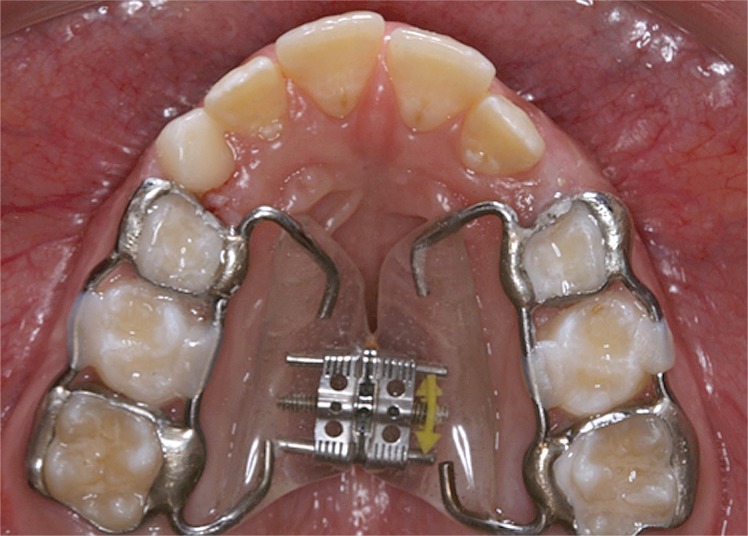
Haas tissue-borne expander.

The RME group comprised 15 children (8 boys and 7 girls, with a mean age of 9.6 years ranging from 7 years and 8 months to 11 years and 6 months) consecutively submitted to RME therapy with a Haas expander (Fig 1). The appliances were standardized with 0.047-in diameter stainless steel wires (Rocky Mountain Orthodontics, Denver, CO, USA) and welded to first molar bands and to deciduous first molars whenever possible. Otherwise, the appliance was bonded and had 11-mm expansion screws (Magnum 600.303.30, Dentaurum, Ispringen, Germany) attached by means of self-curing acrylic resin. All patients were treated by the same operator. Haas activation protocol was used for children under 14 years old.^11^ At delivery, the expander was activated one complete turn (0.8 mm). After initial activation, patient's parents were instructed to activate the expansion screw daily, one-quarter of turn in the morning and in the evening until the required expansion was achieved (according to the individual skeletal deficiency). Progress was weekly monitored during the active phase. Mean screw activation was 7 mm (minimum of 5.6 mm and maximum of 9 mm). The screw was then stabilized with 0.012-in double thread ligature and kept in place passively for 6 months of retention when the expander was removed. Patients were followed up for the next 6 months.

The control group comprised 15 children (10 boys and 5 girls) aged from 7 years and 6 months to 11 years and 4 months (with a mean age of 9.4 years). They met all inclusion criteria and required no orthodontic treatment until the following year.

Oral hygiene instructions were given to all patients. After the 6-month retention period, all patients in need of orthodontic intervention were eventually treated in the Undergraduate Clinics of the Department of Orthodontics and Pediatric Dentistry at the Federal University of Rio de Janeiro.

CBCT scans were taken at onset(T_1_) and one year after RME (T_2_). A similar interval between scans (T_2_-T_1_) was designed for the control group (the mean interval was one year and 3 months for the treated group and one year and 4 months for the control group). All scans were taken using the same machine (i-CAT; Imaging Sciences International, Hatfield, PA, USA) and following a standard protocol (120 KVp, 5 mA, 16x22 cm FOV and 0.4 mm voxel). During scanning, all patients were in maximal intercuspation.

**Figure 2:**
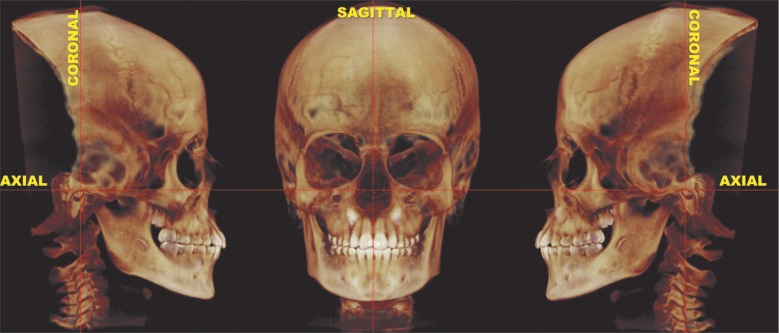
Three-dimensional image of the head after standardization by axial, coronal and sagittal reference planes. Dolphin Imaging(r) 11.5, orientation tool.

Scanning data at T_1_ and T_2_ were exported into DICOM format and imported into the analysis software (Dolphin 3D^(r)^ software, version 11.5, Dolphin Imaging, Chatsworth, CA, USA). After importing, each 3D-volumetric data set was standardized using reference planes.^4^^,^ 12 The axial plane intersected the right and left orbital points as well as the right porion; the coronal plane intersected the left and right porion, perpendicularly to the chosen axial plane; and the sagittal plane intersected the nasion point, perpendicularly to the chosen axial and coronal planes (Fig 2). This procedure was necessary to replicate the positions of the 3D-volumetric data set in T_1_ and T_2_.

All CBCT scans were performed and analyzed by the same examiner in a properly darkened room. The scans were randomly selected and the examiner was blinded for both group and phase of assessment.

**Figure 3:**
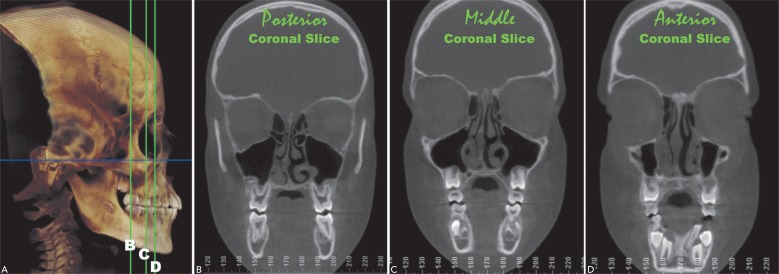
A) 3D visualization of coronal slices used to obtain the transverse measurements. B) Posterior coronal slice; C) Middle coronal slice and D) Anterior coronal slice

**Figure 4:**
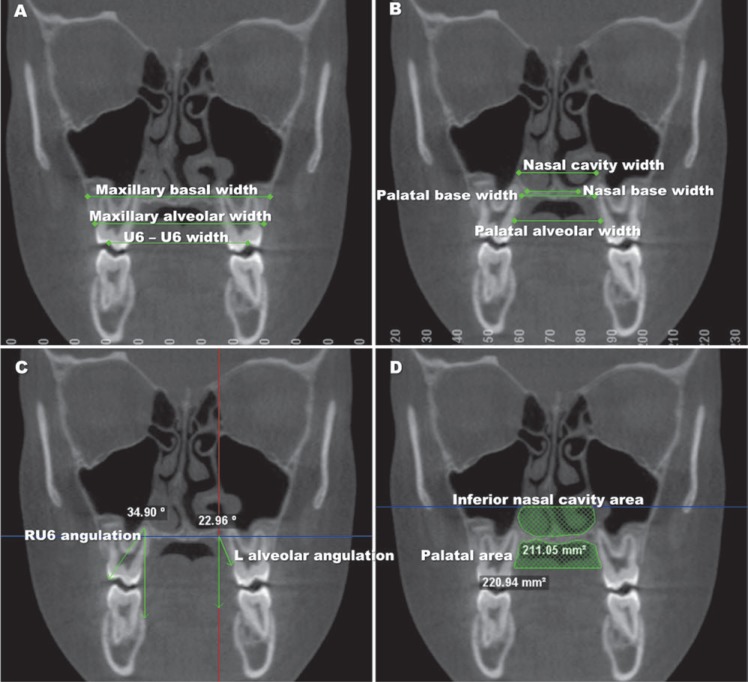
Posterior coronal slices illustrating all measurements taken.

Transverse measurements were performed in three (posterior, middle and anterior) coronal slices. The posterior slice intersected the distal cusp of the right maxillary first molar (U6), the middle slice and anterior slices were located more anteriorly to the posterior slice, 10 mm and 15 mm, respectively (Fig 3). Maxillary first molars (U6) width, right and left U6 angulation, maxillary alveolar width, maxillary basal width, palatal alveolar width, palatal base width, right and left alveolar angulation, palatal area, nasal base width, nasal cavity width and inferior nasal cavity area were obtained on the posterior, middle and anterior slices, except for the first two measurements which were performed on the posterior slice, only. Measurements are described in Table 1 and Figure 4.

**Table 1:** Measurements description.

Measurements	Description
U6-U6 width * (mm)	Distance between the middle fossa of right and left maxillary first molars (Fig 4A).
RU6 angulation * (^o^)	Angle formed between the line intersecting the tip of distobuccal cusp, the palatal root of right maxillary first molar and the sagittal line (Fig 4C).
LU6 angulation *(^o^)	Angle formed between the line intersecting the tip of distobuccal cusp, the palatal root of left maxillary first molar and the sagittal line.
Maxillary alveolar width (mm)	Linear distance between right and left lower and most medial points of the buccal alveolar process (Fig 4A).
Maxillary basal width (mm)	Linear distance between right and left points in the buccal alveolar contours of the maxilla intersecting a parallel line to the axial plane tangent to the lower contours of the nasal cavity (Fig 4A).
Palatal alveolar width (mm)	Linear distance between right and left lower and most medial points of palatal alveolar process (Fig 4B).
Palatal base width (mm)	Linear distance between the most lateral points of the palatal base (Fig 4B).
Right alveolar angulation (^o^)	Angle formed between a line tangent to right palatal alveolar process and the sagittal line.
Left alveolar angulation (^o^)	Angle formed between a line tangent to the left palatal alveolar process and the sagittal line. (Fig 4C).
Palatal area (mm^2^)	Palatal vault cross-sectional area limited by the palatal base, right and left palatal alveolar processes and the line passing by the right and left lower points of the palatal alveolar process. The area was calculated automatically by the software (Fig 4D).
Nasal base width (mm)	Linear distance between right and left most lateral points of the lower contour of the nasal cavity (Fig 4B).
Nasal cavity width (mm)	Linear distance between the most lateral points on the nasal cavity measured parallel to the axial plane (Fig 4B).
Inferior nasal cavity area (mm^2^)	Inferior nasal cavity cross-sectional area limited by the nasal base, the lateral walls of the nasal cavity and a line passing by the upper limit of the lowest nasal concha (Fig 4D).

* Taken on the posterior coronal slice, only.

A statistical software (SPSS, version 17.0, SPSS Inc, Chicago, Ill) was used for statistical analysis with P < 0.05. Intraclass correlation coefficient test (ICC) was applied to assess intraexaminer agreement (95% confidence interval) for all variables. Sixteen CBCT images were randomly selected and all measurements were repeated within a one-week interval. Descriptive statistical analysis (means and standard deviations) was carried out for all variables at T_1_ and T_2_. After assessing normal data distribution (Kolmogorov-Smirnov test), paired Student's t-test was used to identify statistically significant changes in each group. Comparisons of changes over time between treated and control groups were performed by unpaired t -test. Since multiple tests were carried out, Benjamini-Hochberg procedure was applied to avoid type I error (false positive).

## RESULTS

Assessment of intraexaminer agreement test revealed an ICC value ≥ 0.92 for all measurements, thereby demonstrating reproducibility of the CBCT analysis. Mean measurement differences were considered clinically insignificant as they were less than 0.4 mm, 5.3 mm^2^ and 1.3^o^.

Table 2 shows means, standard deviation (SD), results of paired Student's t-test and unpaired t-test for all measurements performed on the posterior, middle and anterior coronal slices for both groups.

**Table 2:** Mean, standard deviation (SD), paired Student's t-test (T1 x T2) and unpaired t-test results (treated x control) for measurements performed on the posterior, middle and anterior coronal slices.

		Treated group				Control group				RME control
		T_1_	T_2_	T_2 _- T_1_	T_1 _x T_2_	T_1_	T_2_	T_2 _- T_1_	T_1 _x T_2_	T_1 _x T_2_
Variables	Slice	Mean ± SD			P-value		Mean ± SD		P-value	P-value
U6-U6 width (mm)	P	43.94 ± 2.6	46.71 ± 2.6	2.76 ± 1.1	0.000***	47.10 ± 2.7	47.11 ± 2.7	0.01 ± 1.2	0.974	0.000***
RU6 angulation (degrees)	P	35.85 ± 4.4	30.76 ± 6.4	-5.09 ± 4.7	0.002**	36.70 ± 4.9	35.44 ± 3.7	-1.26 ± 3.6	0.390	0.035*
LU6 angulation (degrees)	P	34.54 ± 4.6	30.85 ± 6.6	-3.69 ± 5.0	0.021*	37.57 ± 3.3	36.54 ± 4.6	-1.02 ± 4.0	0.549	0.154
Maxillary alveolar width (mm)	P	54.28 ± 2.8	59.03 ± 3.5	4.74 ± 2.1	0.000***	58.15 ± 2.3	58.11 ± 2.9	-0.03 ± 1.4	0.953	0.000***
Maxillary alveolar width (mm)	M	49.35 ± 2.4	53.83 ± 2.9	4.48 ± 1.4	0.000***	53.97 ± 2.6	53.57 ± 3.3	-0.39 ± 0.9	0.271	0.000***
Maxillary alveolar width (mm)	A	44.52 ± 3.3	49.06 ± 2.8	4.53 ± 2.7	0.000***	50.54 ± 3.2	50.16 ± 2.7	-0.37 ± 1.4	0.561	0.000***
Maxillary basal width (mm)	P	60.37 ± 2.9	63.02 ± 3.6	2.65 ± 1.5	0.000***	62.29 ± 2.3	63.53 ± 2.7	1.24 ± 0.9	0.000***	0.010*
Maxillary basal width (mm)	M	58.53 ± 12.1	57.91 ± 10.6	-0.62 ± 4.6	0.653	62.86 ± 8.2	59.61 ± 7.3	-3.25 ± 2.2	0.000***	0.077
Maxillary basal width (mm)	A	42.32 ± 7.1	43.50 ± 6.4	1.18 ± 2.9	0.164	48.81 ± 5.2	45.75 ± 4.0	-3.06 ± 2.4	0.000***	0.000***
Palatal alveolar width (mm)	P	29.97 ± 2.6	33.88 ± 3.0	3.91 ± 1.2	0.000***	32.68 ± 2.3	33.26 ± 2.2	0.58 ± 0.9	0.112	0.000***
Palatal alveolar width (mm)	M	27.26 ± 3.1	30.50 ± 3.8	3.24 ± 2.6	0.000***	29.99 ± 2.3	29.48 ± 1.9	-0.50 ± 1.4	0.392	0.000***
Palatal alveolar width (mm)	A	23.69 ± 2.4	27.38 ± 2.7	3.68 ± 2.2	0.000***	28.01 ± 2.2	27.02 ± 2.2	-0.99 ± 2.2	0.254	0.000***
Palatal base width (mm)	P	22.05 ± 3.7	27.16 ± 2.7	5.10 ± 3.9	0.000***	25.76 ± 2.7	27.11 ± 2.4	1.35 ± 2.7	0.215	0.011*
Palatal base width (mm)	M	16.33 ± 2.9	18.69 ± 4.3	2.36 ± 3.1	0.020*	20.10 ± 3.1	20.18 ± 3.1	0.08 ± 3.4	0.984	0.102
Palatal base width (mm)	A	14.97 ± 2.3	17.07 ± 5.2	2.09 ± 4.8	0.148	17.61 ± 2.6	16.81 ± 3.6	-0.80 ± 3.7	0.571	0.106
R alveolar angulation (degrees)	P	20.51 ± 6.5	19.37 ± 4.5	-1.14 ± 6.3	0.546	35.80 ± 71.8	17.71 ± 5.7	-18.09 ± 72.4	0.550	0.441
L alveolar angulation (degrees)	P	17.44 ± 3.7	18.81 ± 5.3	1.36 ± 5.9	0.440	34.12 ± 58.0	18.45 ± 5.3	-15.67 ± 58.1	0.545	0.328
R alveolar angulation (degrees)	M	28.25 ± 6.2	27.90 ± 8.6	-0.35 ± 8.2	0.870	22.71 ± 6.1	24.57 ± 10.2	1.86 ± 8.2	0.587	0.510
L alveolar angulation (degrees)	M	28.11 ± 7.62	25.86 ± 6.7	-2.25 ± 6.8	0.259	25.25 ± 5.6	24.79 ± 6.1	-0.45 ± 4.2	0.804	0.447
R alveolar angulation (degrees)	A	27.28 ± 8.1	31.55 ± 9.2	4.26 ± 10.1	0.156	23.57 ± 5.3	24.32 ± 7.1	0.75 ± 6.1	0.816	0.327
L alveolar angulation (degrees)	A	27.05 ± 9.4	27.56 ± 9.6	0.51 ± 9.2	0.859	25.90 ± 5.6	26.89 ± 5.4	0.98 ± 5.1	0.618	0.864
Palatal area (mm^2^)	P	266.45 ± 37.5	326.76 ± 51.3	60.31 ± 24.0	0.000***	281.66 ± 47.5	314.02 ± 54.8	32.35 ± 27.7	0.000***	0.079
Palatal area (mm^2^)	M	244.63 ± 41.6	288.05 ± 59.4	43.42 ± 40.5	0.002**	277.68 ± 46.5	285.99 ± 44.3	8.30 ± 36.2	0.561	0.035*
Palatal area (mm^2^)	A	182.34 ± 46.9	209.66 ± 59.5	-172.50 ± 46.1	0.059	259.80 ± 45.7	230.99 ± 25.1	-248.65 ± 45.5	0.062	0.000***
Nasal base width (mm)	P	21.10 ± 2.3	23.92 ± 2.8	2.81 ± 1.9	0.000***	23.27 ± 2.1	23.09 ± 1.6	-0.17 ± 1.5	0.799	0.000***
Nasal base width (mm)	M	18.75 ± 2.4	20.81 ± 2.5	2.05 ± 2.2	0.006**	20.16 ± 2.4	19.73 ± 2.0	-0.43 ± 1.2	0.392	0.003**
Nasal base width (mm)	A	15.29 ± 4.4	17.87 ± 2.8	2.57 ± 4.0	0.039*	17.75 ± 2.6	17.86 ± 3.3	0.11 ± 2.2	0.930	0.080
Nasal cavity width (mm)	P	25.68 ± 3.6	27.79 ± 4.2	2.11 ± 1.0	0.000***	26.41 ± 3.0	26.97 ± 2.9	0.55 ± 0.7	0.047*	0.000***
Nasal cavity width (mm)	M	26.67 ± 3.2	28.28 ± 3.4	1.60 ± 1.8	0.000***	27.91 ± 2.9	28.28 ± 2.8	0.37 ± 0.5	0.070	0.003**
Nasal cavity width (mm)	A	24.71 ± 3.7	26.48 ± 2.8	1.77 ± 2.6	0.030*	26.03 ± 2.7	25.46 ± 2.7	-0.57 ± 1.0	0.114	0.007**
Inferior nasal cavity area (mm^2^)	P	270.78 ± 69.2	314.88 ± 90.0	44.10 ± 40.6	0.002**	264.84 ± 70.0	302.31 ± 78.6	37.46 ± 27.3	0.000***	0.622
Inferior nasal cavity area (mm^2^)	M	394.31 ± 78.0	435.63 ± 88.0	41.31 ± 55.8	0.020*	423.72 ± 92.9	475.49 ± 102.9	51.76 ± 32.5	0.000***	0.571
Inferior nasal cavity area (mm^2^)	A	375.20 ± 92.2	429.09 ± 107.4	53.89 ± 90.9	0.055	428.10 ± 124.8	420.56 ± 119.3	-7.53 ± 112.2	0.909	0.153

P: posterior slice; M: middle slice; A: anterior slice

* P < 0.05

** P < 0.01

*** P < 0.001.

Maxillary first molar width was statistically significant greater (2.76 mm) one year after RME in comparison to the control group, even though U6 tipping had decreased significantly (RU6, -5.09^o^ and LU6, -3.69^o)^.

Maxillary alveolar width, basal width, and palatal alveolar width were significantly increased in the treated group compared to the control group in all coronal slices. Palatal base width was statistically significant increased in the treated group compared to the control group only in posterior slices. Alveolar angulations did not change significantly in either group. Palatal area was significantly greater in the treated group, only.

Regarding nasal cavity width and base width, the treated group presented a significant increase compared to the control group in all coronal slices. However, there were no significant changes in the inferior nasal cavity compared to the control group (P > 0.05).

## DISCUSSION

This prospective controlled clinical study assessed the transverse changes promoted by RME on the nasomaxillary complex one year after treatment with Haas expander. This expander was used passively for a 6-month period as retention. No subsequent orthodontic therapy using fixed or removable appliances was performed.

One year after RME, intermolar width was 2.75 mm greater in comparison to the control group, even though tipping had decreased significantly (RU6, -5.09^o^ and LU6, -3.69^o)^. Patients with similar skeletal maturation (stages CS1 - CS3) were assessed by means of posteroanterior cephalograms and revealed an increase of 2.7 mm in maxillary intermolar width, which was significantly greater in comparison to the control group 5 years after RME (Haas expander) and nonextraction edgewise treatment.^13^ Lima et al^14^ used dental casts to assess short (one year) and long-term (5 years) effects of RME associated with Haas appliance during mixed dentition. Results revealed an average increase of 5.64 mm in maxillary arch width one year after treatment, with a relapse of 1.13 mm 5 years after treatment. However, no control group was considered. This greater increase observed in that study may be attributed to differences in the retention protocol. They used the expander passively for an average period of 5 months and after its removal, a retention plate was used for at least one year. In the present study, no other retainer was used after removing the expander (after 6 months for retention). Therefore, longer retention periods should be considered if wider maxillary arch is required.

RME has been the treatment of choice for many orthodontists aiming to correct skeletal maxillary constriction in growing patients.^4^ Maxillary alveolar and basal width significantly increased in the treated group in all coronal slices. The mean increase of alveolar width was greater than 4.0 mm for all slices in the treated group. Palatal expansion resulted in widening of the maxilla, both in the posterior and anterior portions.

This fact may be confirmed by the significantly greater palatal alveolar width changes found in the treated group (P: 3.91 mm; M: 3.24 mm; A: 3.68 mm) compared to the control group (P: 0.58 mm; M: 0.50 mm; A: -0.99 mm). Nevertheless, palatal base width changes were significantly greater in the posterior slice, only (5.0 mm). Pangrazio-Kulbersh et al^15^ also analyzed CBCT scans and found an increase of 3.2 mm (P = 0.001) in posterior palatal alveolar width in the group tooth-borne expander with bands, whereas the group with bonded tooth-borne expander had an increase of 1.78 mm (P = 0.001). Tipping was observed in both groups. On the other hand, assessment was performed immediately after the removal of the expander (6-month retention period) and some relapse was expected. The greater increase observed one year after RME may be explained by the tissue-borne expander (Haas) used in our study.

Successful results might probably be attributed to the choice of the expander. The Haas expander screw is immersed in acrylic pads in contact with the palate, providing not only more anchorage during RME, but also more stability of the palatal alveolar process changes in the retention period. No significant changes were found in alveolar angulation, thereby suggesting that the RME protocol induced normal development. Modifications in palatal widths may be explained by changes in palatal shape: from triangular to square.^16^

Muchitsch et al^17^ quantified changes in the palatal vault area 6 months after RME with bonded expanders. Their study used digital dental casts. The mean increase of permanent first molar, deciduous second molar and deciduous canine cross-sectional areas was 20.46 mm^2^, 21.39 mm^2^ and 20.39 mm^2^, respectively. No control group was considered. In our study, palatal area changes in the treated group were greater than in the control groups (27.96 mm^2^, 35.12 mm^2^ and 76.15 mm^2^ in posterior, middle and anterior slices, respectively). Importantly, the palatal cross sectional areas determine the amount of space available for the tongue, and for this reason, affect its physiological function. Ozbek et al^18^ observed higher tongue posture after RME in children with no respiratory disturbances and stated that this spontaneous alteration in tongue posture may be related to stability of RME.

A recent systematic review assessing the long-term effects of RME on the airway dimensions of growing children found moderate evidence of stability of transverse increase promoted by RME within 11 months after treatment.^19^ Two studies reported an average increase in nasal cavity width of 2.2 mm and 4.16 mm 11 months^20^ and 5 years^5^ after RME. Both studies were performed with posteroanterior cephalograms. One year after RME, our study found an increase of 2.81 mm, 2.05 mm and 2.57 mm in nasal base width (posterior, middle and anterior slices, respectively) and of 2.11 mm, 1.60 mm and 1.77 mm in nasal cavity width (posterior, middle and anterior slices, respectively). These increases were significantly higher in comparison to the control group. In assessing the increase in the inferior nasal cavity area, no significant difference was observed between treated and control groups. The only CBCT study^21^ included in this systematic review^19^ assessed changes in retropalatal and retroglossal airways volume after RME. They found no significant difference between treated and control groups; however, maxillary width increased significantly in the RME group. The authors stated that a possible bias in this retrospective study was the absence of control over tongue position when the CBCT scans were taken. In addition, the software used in this study was considered highly reliable, but with poor accuracy.^22^ Our findings suggest that RME also expands the nasal cavity, in which case widening remains stable one year after treatment.

The literature indicates that RME produces an average of 50% skeletal and 50% dentoalveolar changes.^23^ Our results showed that skeletal effects were greater than dentoalveolar effects one year after treatment. All patients were at prepubertal stages of skeletal maturation.^8^ RME treatment timing proves to highly influence treatment effects. When RME is performed in prepubertal subjects, it produces more skeletal transverse changes than in postpubertal subjects.^13^

CBCT is a scanning technique that provides higher resolution measurements of the transverse dimensions of the skeletal structure. Nevertheless, the everyday use of CBCT is not recommended in orthodontic practice, since conventional radiographs deliver lower radiation doses to patients. Even so, some orthodontic patients require posteroanterior and lateral cephalograms, as well as panoramic, periapical, occlusal or bitewing radiographs. According to Gibbs,^24^ the effective dose for a full-mouth radiographic survey and the sum of the effective doses for these images are similar or even higher than that of the CBCT. Thus, CBCTs may be the best choice in some cases. When 3D imaging is required in orthodontic practice, CBCT should be preferred over multi-slice CT.^25^ This study used CBCT scans because 3D evaluation was also performed, as stated previously.^4^^,^ 12 ^,^^26^^,^^27^

Therefore, our study rejected the tested hypothesis that there were no differences in the transverse changes of the nasomaxillary complex comparing patients submitted to RME and untreated subjects. One year after RME, the maxillary, palatal and nasal transverse dimensions were significantly increased when compared to the control group. Long-term clinical response demonstrated the efficacy and stability of the RME protocol used to achieve nasomaxillary transversal increase.

## CONCLUSIONS

RME significantly increased* molar, maxillary, palatal and nasal *widths in comparison to the control group, thereby demonstrating stability one year after treatment.
